# Profil diagnostique et évolutif du myélome multiple au Sénégal: étude monocentrique de 2005 à 2016

**DOI:** 10.11604/pamj.2017.27.262.13164

**Published:** 2017-08-08

**Authors:** Seynabou Fall, Fatma Dieng, Coumba Diouf, Boundia Djiba, Awa Cheikh Ndao, Fatou Samba Diago Ndiaye

**Affiliations:** 1 Unité d’Hématologie Clinique de l’Hôpital Aristide Le Dantec, Dakar, Sénégal; 2 Service de Rhumatologie de l’Hôpital Aristide Le Dantec, Dakar, Sénégal; 3 Service de Médecine Interne de l’Hôpital Aristide Le Dantec, Dakar, Sénégal

**Keywords:** Myélome multiple, Index Staging Sytem, traitement innovant, survie, Multiple myeloma, index staging system, innovative treatment, survival

## Abstract

**Introduction:**

Les thérapeutiques innovantes du myélome multiple sont peu accessibles en Afrique subsaharienne. Le but de cette étude est de décrire les particularités diagnostiques et évolutives observées dans notre pratique de prise en charge des myélomateux.

**Méthodes:**

Une étude rétrospective (2005 - 2016) descriptive à visée analytique, mené à l’hôpital Le Dantec (Sénégal) a concerné les myélomateux inclus selon les critères de l’International Myeloma Working Group (2003, 2014).

**Résultats:**

Ont été colligés 136 dossiers (69 hommes, 67 femmes) de patients d’âge moyen 59 ans ± 10,1 ans et qui ont un âge inférieur à 65 ans dans 69,1% des cas. Les signes révélateurs ont été des douleurs osseuses (96,3%), une insuffisance rénale (36,8%), une infection (23,5%), une fracture pathologique (17,6%), une compression médullaire (16,9%), et une hypercalcémie maligne (16,2%). L’isotype a été IgG dans 61,3% des cas et Kappa dans 65% des cas. Les malades ont été classés stade III (59,4%) et I-II (40,6%) de l’index staging system. Sous traitement conventionnel (Méphalan-Prédnisone: 67,6%, innovant: 5,9%), la survie médiane a été de 20 mois (1-78 mois). La survie est meilleure, en l’absence de complications neurologiques, infectieuses et au score I-II de l’Index Staging System.

**Conclusion:**

Dans notre étude, le myélome multiple est fréquemment diagnostiqué avant 65 ans, au stade de forte masse tumorale. La survie globale peut être améliorée par un dépistage précoce et un accès aux thérapeutiques adéquates.

## Abstract (en)

**Introduction:**

Accessibility to innovative multiple myeloma therapies is limited in sub-Saharan Africa. This study aimed to describe the diagnostic and evolutionary features observed during treatment of our patients with myeloma.

**Methods:**

We conducted a retrospective, descriptive, analytical study (2005 - 2016) of patients with myeloma included in the study based on International Myeloma Working Group (IMWG) Criteria (2003,2014) at the Hopital Aristide Le Dantec (Senegal).

**Results:**

We collected data from 136 medical records (69 men, 67 women) of patients with an average age of 59 years ± 10.1 years, who were less than 65 years of age in 69.1% of cases. Tell-tale signs included bone pain (96.3%), renal failure (36.8%), infection (23.5%), pathological fracture (17.6%), spinal cord compression (16.9%) and malignant hypercalcaemia (16.2%). Isotopic antiglobulin test showed that anti-IgG could be detected in 61.3% of cases and Kappa in 65% of cases. Patients were classified stage III (59.4%) and I-II (40.6%)of the index staging system. The median survival of patients under conventional traitement (Méphalan-Prédnisone: 67.6%, innovative: 5.9%) was 20 months (1-78 months). Survival rates are better in the absence of neurological and infectious complications and for patients with score I-II of the index Staging System.

**Conclusion:**

In our study, multiple myeloma was frequently diagnosed before age 65, at advanced stage of tumor mass. Early detection and access to adequate therapies could improve overall survival.

## Introduction

Le myélome multiple (MM) est une dyscrasie plasmocytaire médullaire maligne, liée à la sécrétion d’une immunoglobuline monoclonale. C’est la plus fréquente des gammapathies malignes [1]. Les manifestations polymorphes, sont la base de critères diagnostiques qui ont été réactualisés en 2014 [2]. Ceci dans un contexte d’innovations, d’avancées thérapeutiques qui ont amélioré la survie de patients atteints du MM qui reste une affection incurable. En Afrique subsaharienne, se pose la problématique de l’accessibilité aux médications innovantes; et à notre connaissance, peu de publications ont évalué les aspects thérapeutiques du MM. Notre but est de décrire les particularités diagnostiques et évolutives observées dans notre pratique de prise en charge des myélomateux.

## Méthodes

Une étude rétrospective, descriptive à visée analytique, a été menée de Janvier 2005 à Décembre 2016 au service de Médecine Interne du CHU Aristide Le Dantec (Sénégal), qui abrite l’Unité d’Hématologie Clinique et de Rhumatologie. Ont été inclus les dossiers de patients suivis pour un MM retenu selon les critères de l’International Myeloma group (IMWG) datant de 2003 et de 2014 [2, 3] en fonction du diagnostic avant ou après 2014. Ont été analysés les caractéristiques de la population d’étude: âge, genre, les symptômes, les données de l’IMWG [2, 3]: profil immunochimique protéique, médullogramme, signes CRAB (hypercalcémie, atteinte rénale, anémie, lésions osseuses); le pronostic: Salmon Durie [4], International Staging System (ISS) [5] et les modalités évolutives. Au plan thérapeutique, l’autogreffe indiquée chez les malades de moins de 65 ans, n’est pas disponible dans notre pratique, mais certains ont pu en bénéficier en dehors du Sénégal. Ainsi, les protocoles en fonction des ressources financières ont été l’association Melphalan-Prédnisone (MP) ou Alexanian, Méphalan-Prédnisone-thalidomide(MPThal), Vélcade-Thalidomide-Dexaméthasone (VTDex). Nous avons défini comme traitement innovant, les protocoles MPthal, VTDex et l’autogreffe. Au plan évolutif, ont été analysées les réponses thérapeutiques [6]: rémission complète (RC), partielle (RP), réponse partielle de bonne qualité (RPBQ), absence de réponse (perte de réponse, rechute, la stabilité). L’analyse de la survie a concerné les patients suivis (vivants, décès) sans les perdus de vue. La saisie des données, les analyses statistiques (moyenne, médiane, écart type, test de Student) et la courbe de Kaplan Meier ont été effectués avec le logiciel SPSS20.0.

## Résultats

**Figure 1:**
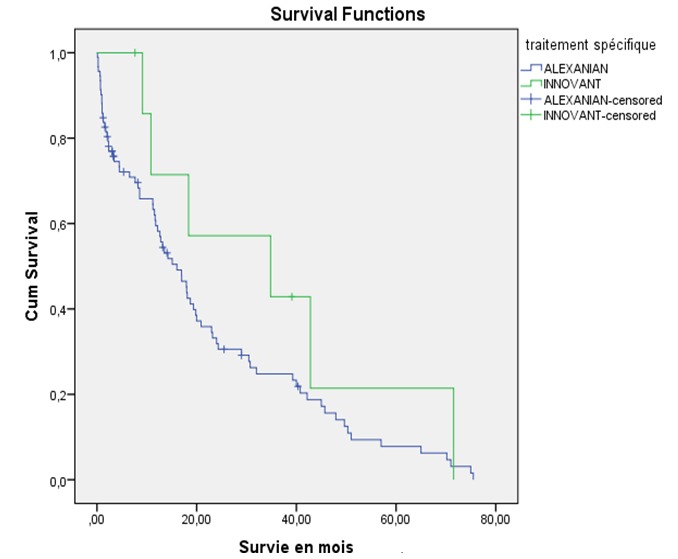
Survie selon le traitement conventionnel chez les myélomateux à l’hôpital Le Dantec (2005 - 2016)

**Figure 2:**
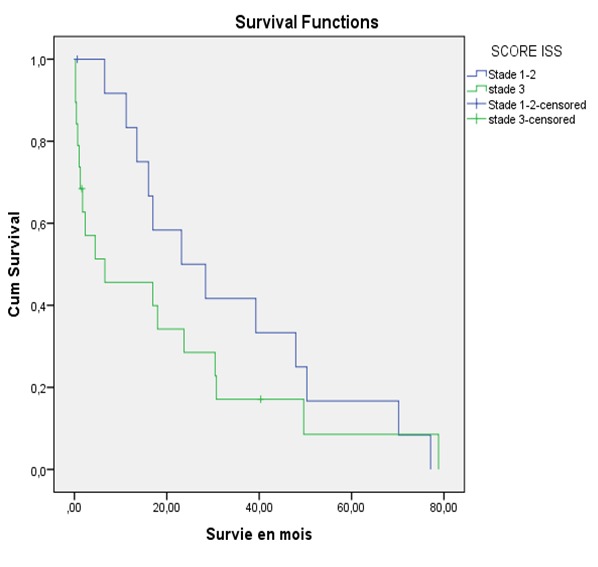
Survie selon le score Index Staging System chez les myélomateux à l’hôpital Le Dantec (2005 -2016)

Notre étude a concerné 136 dossiers de malades dont les caractéristiques sociodémographiques (Tableau 1) ont été un âge moyen de 58,8 ± 10 ans, un âge de moins de 65 ans dans 69,1% des cas et un sex- ratio de 1,09. Le délai diagnostic moyen entre l’apparition des premiers signes et la consultation a été de 10 ± 7,9 mois. . Les signes cliniques (Tableau 1) à l’admission ont été des douleurs osseuses (96,3%), une altération de l’état général (86,7%), un plasmocytome osseux (11 cas) et des complications. Ces dernières sont représentées par l’altération de la fonction rénale (50 cas dont 34 cas d’insuffisance rénale fonctionnelle), l’infection (23,5%), la fracture pathologique périphérique (17,6%), la compression médullaire (16,9%) et l’hypercalcémie maligne (16,4%). A l’analyse de la sécrétion protéique, le taux moyen de protéines totaux est de 89 ± 22,6 g/l. Le protidogramme (Tableau 1) a montré un pic gammaglobuline dans 68,9% des cas à côté d’autres profils. L’immunofixation des protéines sériques (Tableau 1) quand elle est effectuée, a mise en évidence une sécrétion d’IgG (49 cas), IgA (11 cas), IgE (1cas) à chaine légère (12 cas) et double IgG-IgA (4 cas). Il s’agissait d’un MM à chaine légère Kappa dans 65% des cas ou lambda dans 35% des cas. L’infiltration plasmocytaire était significative dans 71% des cas et au-delà de 60% dans 15,4% des cas (Tableau 1). Les signes CRAB sont présents chez tous les malades (Tableau 1) qui ont 3 signes et plus dans 62,5% des cas ou 1 à 2 signes dans 37,5% des cas. Les modifications radiologiques (tableau 1) en dehors des géodes multiples (88,1%), ont été une déminéralisation diffuse (55,9%), une fracture périphérique (17,6%) et un tassement vertébral (15,4%). Une épidurite était à l’origine de la compression médullaire dans 1 cas. Au plan pronostic, la classification de Salmon Durie a été déterminée chez 127 malades qui ont été au stade III dans 77,9% des cas, ou I-II dans 16,5% des cas. L’ISS est disponible dans 32 dossiers de malades classés stade III (59,4%) ou I-II (40,6%). Sur le plan thérapeutique, les malades ont été sous biphosphonates (44,8%) et médication spécifique (73,5%) composé de MP (92 cas) et de traitement innovant: VTDex (3 cas), MP Thal (2 cas), autogreffe de moelle osseuse (3 cas). A la fin de l’étude, 55 malades vivants ont obtenu une RPBQ (1 cas), une RC (2 cas), une RP (16 cas), une absence de rémission (progression : 25 cas, rechute: 6 cas, stable : 5 cas), et 24 autres sont décédés. Aucune RC n’est atteinte sous MP. Nous avons répertorié 57 cas de perdu de vue. La médiane de survie a été de 20 mois (0,1-78 mois). L’analyse de la courbe de Kaplan Meier sous traitement innovant versus (Vs) MP, a montré une survie cumulée à 24 mois de 60% Vs 30% (p : 0,07) (Figure 1). Selon les scores ISS III Vs I-II, la survie à 24 mois a été de 30% Vs 50% (p: 0,021) (Figure 2). L’analyse univariée de médiane de survie (Tableau 2) en l’absence ou non de complications a montré une durée de : 23,7 Vs 18,7 mois pour la néphropathie (p: 0,03), 23 Vs 14 mois pour les infections (p : 0,05), 23 mois Vs 16 mois pour la compression médullaire (p: 0,00), 26,53 Vs 25,7 mois et pour l’hypercalcémie maligne (0,013). La survie médiane est de 23,1 Vs 23 mois chez les malades qui ont 2 signes CRAB ou plus 2(p: 0,29). Selon l’ISS I-II Vs III, la médiane de survie est de 28,4 Vs 20,4 mois (p: 0,021). Selon l’usage ou non de traitement innovant, la survie médiane a été 19,8 Vs 20,3 mois (p: 0,05).

**Tableau 1:** Données diagnostiques des myélomateux à l’hôpital Le Dantec (2005 - 2016)

Paramètres	n (%)
**Démographie** (N=136)	
Genre : H / F	69 (50,7) / 67 (49,3)
Age : ≤ 65 ans / ˃65 ans	94 (69,1) / 42 (30,9)
**Signes révélateurs** (N=136)	
Douleurs osseuses	131 (96,3)
AEG	118 (86,7)
Altération fonction rénale	50 (36,8)
Infection	32 (23,5)
Fractures périphériques pathologiques	24 (17,6)
Compression médullaire lente	23 (16,9)
Hypercalcémie maligne	22 (16,2)
**Biologie** (N=136)	
Hémoglobine < 12 g/dl	113 (83,1)
Calcémie ˃105 mg/l	68 (50)
Créatininémie ˃ 20 mg/l	50 (36,7
**Médullogramme** (N= 128)	
plasmocytose ˃ 10 %	91 (71,1)
˃ 60%	21 (16,4)
**Electrophorèse protéines sériques** (N=119)	
pic gamma globuline	82 (68,9)
pic béta globuline	18 (15,1)
pic inflammatoire	5 (4,2)
double pic béta/gamma globuline	5 (4,2)
hypo gamma globuline	9 (7,6)
**Immunofixation protéines sériques** (N=80)	
IgG	49 (61,3)
IgA	11 (13,8)
IgE	1 (1,3)
Chaines légères	12 (15)
Biclonale (IgA/IgG)	4 (5)
Chaines légères libres	3 (3,8)
kappa	52 (65)
lambda	28 (35)
**Radiologie** (N=136)	
Géodes	119 (88,1)
Déminéralisation diffuse	76 (55,9)
Tassement vertébrale	21 (15,4)
Tumeur plasmocytaire	11 (8,1)
N: effectif total; n: effectif atteint ; %: pourcentage; H/F: homme/femme; AEG: altération de l’état général; CRP: C-réactive protéine; IgG: immunoglobuline G; IgA : immunoglobuline A; IgE : immunoglobuline E	

**Tableau 2:** Analyse univariée de la survie médiane des myélomateux à l’hôpital Le Dantec (2005 -2016)

Paramètres	(n)	Survie médiane (mois)	P
**Insuffisance rénale**			
Oui	(29)	23,7	0, 03
Non	(50)	18,7	
Infection			
Oui	(21)	14	0,05
Non	(58)	23	
**Fracture pathologique**			
Oui	(13)	16	0,4
Non	(66)	23	
**Compression médullaire**			
Oui	(24)	16	0,00
non	(55)	23	
**Hypercalcémie maligne**			
Oui	(12)	25,7	0,07
Non	(66)	11,7	
**Nombre de signes CRAB**			
1-2	(31)	23,1	0,29
3-4	(48)	23	
**Plasmocytose médullaire**			
Inférieure à 60%	(57)	25,5	0,09
Supérieure à 60%	(21)	13,2	
Salmon Durie			
Stade I-II	(13)	23,1	0,07
Stade III	(44)	18,7	
n: effectif suivi; p: test de Student			

## Discussion

Notre étude sur 136 myélomateux fait partie des rares grandes séries rapportées en Afrique noire subsaharienne [7, 8, 9–13]. Ce large recrutement de nos malades est lié à la proximité des services prise en charge au CHU Le Dantec. Le MM est observé près de la soixantaine, dans les publications africaines [7–9]. L’âge moyen dans notre étude (58,8 ans ± 10 ans), est quasi similaire à celui rapporté au Nigéria [7] (58,8 ± 11,2 ans) et en Egypte [8] (58,5 ans). Le MM est fréquent chez les sujets jeunes de moins de 65 ans qui représentent plus de la moitié dans notre étude et celle de Diebkéle AT et al en Côte d’Ivoire [10]. De même, Kakpovi et al [11] observaient que 40 % des myélomateux reçu en rhumatologie au Togo, avaient moins de 50 ans. Cependant, le MM reste rare avant 40 ans [8–12]. La prédominance est particulièrement masculine [7–9]. Le délai diagnostique long dans nos régions serait imputable à la difficulté d’accès aux structures adaptées à la prise en charge [11, 13]. Les données du médullogramme de 2014 [3], sont accessibles à nos pratiques et permettraient un diagnostic et un traitement précoce dans nos régions. D’autant plus que les symptômes du MM sont polymorphes, mais restent dominées par la douleur osseuse dans notre travail (93,3%) et d’autres publications qui rapportent des fréquences dépassant 80% [7, 11, 14]. L’altération de l’état général, est fréquente dans nos régions est en parti liée au retard diagnostique [11, 13]. Ainsi, les complications souvent révélatrices du MM [7, 8, 12]. Dans notre série, il s’agissait plus d’une insuffisance rénale (36,8%) décrite dans 20-40% des cas [12, 15]. Dans nos régions, l’usage de médications traditionnelles [16] potentiellement néphrotoxique est un facteur surajouté à la survenue de l’altération rénale au cours du MM. Les autres complications infectieuses, ostéo-neurologiques et métaboliques ne sont pas exceptionnelles [7, 8, 12]. Le profil immunochimique est dominé par l’isotype IgG de type Kappa dans notre étude et dans la littérature [7, 12, 14]. Le MM est souvent diagnostiqué aux stades avancés de forte masse tumorale [7, 8, 11], tel noté dans notre série avec plus de la moitié des malades reçus au stade III de l’ISS et de Salmon et Durie. Au plan thérapeutique, les agents innovants sont peu accessibles en Afrique où le protocole Alexanian est une réalité, qui ne donne pas de RC [7–12]. Dans ce contexte, peu de publications sont axées au traitement et la survie [7, 8, 12]. La médiane de survie observée dans notre série (20 mois) est légèrement en deçà de celui décrit par les auteurs en Tunisie (26 mois) [12]. Une survie plus élevée est publiée au Nigéria (39,7 mois) [7] et en Egypte (37,5 mois) [8]. Ces différences sont liées aux possibilités thérapeutiques. Ainsi, les auteurs ont noté que l’usage de l’autogreffe de moelle osseuse associée à des agents innovant a amélioré la survie globale qui est de 66% à 5 ans [16]. Ce constat est fait aussi chez nos malades, mais L’absence de test significatif est un biais lié à l’effectif limité des malades qui ont reçu les nouvelles thérapeutiques. Hormis, la nature thérapeutique, la survie dépend des complications au diagnostic. Dans notre étude, l’atteinte rénale est associée à une baisse significative de la survie médiane. Ce constat est aussi fait par Kim K et al [17] qui rapportent une médiane de survie de de 52 Vs 31 mois (p < 0,001) selon l’existence ou non de néphropathie au diagnostic. A côté de la néphropathie, l’infection et la compression médullaire lente sont associées à un risque péjoratif de la survie, dans notre série.

## Conclusion

Dans notre étude, le myélome multiple est plus diagnostiqué à un stade avancé de forte masse tumorale, chez des sujets jeunes qui ont peu accès à la greffe de moelle et à la chimiothérapie innovante. Les complications au diagnostic et les stratégies thérapeutiques sont des facteurs identifiés de survie. Améliorer la survie nécessite un dépistage précoce et la disponibilité des moyens thérapeutiques adaptés.

### Etat des connaissances actuelle sur le sujet

- Le myélome multiple est une réalité en Afrique, à travers diverses publications hospitalières;
- Le diagnostic est tardif: les manifestations au diagnostic sont dominées par les symptômes osseux;
- En Afrique subsaharienne, la survie est limitée car les médications innovantes et l’autogreffe ne sont pas disponibles.

### Contribution de notre étude à la connaissance

- L’effectif de notre population d’étude qui est une des rares grandes séries sur le myélome multiple en Afrique de l’Ouest subsaharienne;
- La fréquence élevée des complications au diagnostic de la maladie;
- L’impact négatif sur la survie des complications présentes au diagnostic, d’où la nécessité d’un dépistage précoce des sujets à risque.

## Conflits d’intérêts

Les auteurs ne déclarent aucun conflit d'intérêt.
